# Use of a transparent cap with a slit facilitates endoscopic injection sclerotherapy of esophageal varices

**DOI:** 10.1055/a-2113-9694

**Published:** 2023-07-13

**Authors:** Kuniyo Gomi, Hiroshi Takahashi, Erika Yoshida, Misako Tohata, Yorimasa Yamamoto, Masatsugu Nagahama

**Affiliations:** Division of Gastroenterology, Showa University Fujigaoka Hospital, Yokohama City, Japan

Prophylactic treatment for bleeding is indicated for esophageal varices if the varices are F2 or greater, or if the red-color sign is positive 1 2 . In Japan, endoscopic treatment is the first choice of treatment for bleeding esophageal varices. Endoscopic injection sclerotherapy (EIS) includes the “EO method,” where 5 % ethanolamine oleate (EO) is injected intravascularly, and the “AS method,” where 1 % aethoxysklerol is injected extravascularly. The EO method involves endoscopic varicealography during injection sclerotherapy, with the esophageal varices and their supply tracts being occluded under fluoroscopic guidance; however, endoscopic puncture of esophageal varices and the holding of the needle tip in the blood vessel require advanced techniques. Thin varices are difficult to puncture and, even after they are punctured successfully, respiratory fluctuations, esophageal peristalsis, and the patient’s body movements can dislodge the needle. Therefore, we developed a transparent cap with a slit to facilitate puncture and needle fixation in this situation ( Fig. 1 ).

**Fig. 1:**
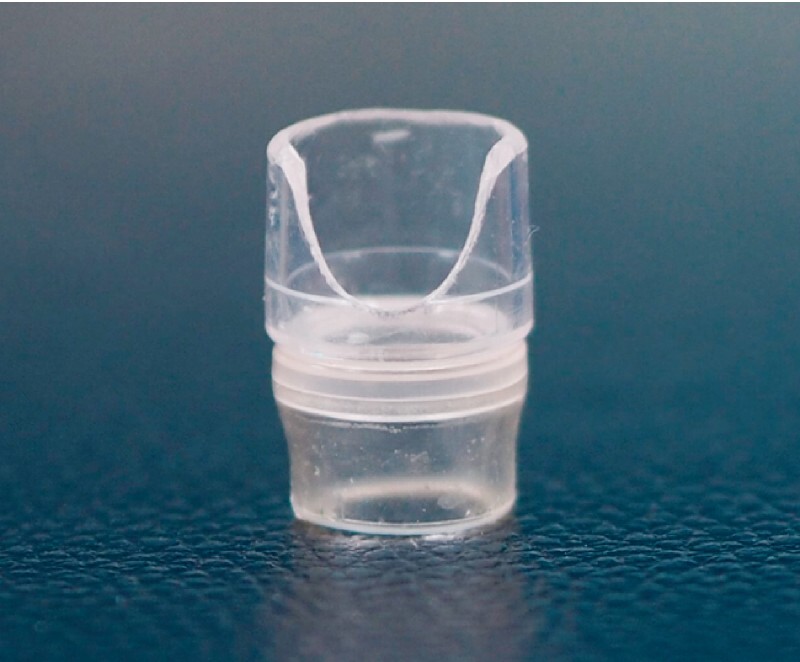
Photographs showing the transparent cap with a slit that facilitates endoscopic injection sclerotherapy.

A 70-year-old woman with liver cirrhosis had esophageal varices (Lm, F2, Cb, RC3). We therefore decided to perform endoscopic injection sclerotherapy using 5 % EO. First, a fixation balloon and a transparent cap with a slit were attached to an endoscope ( Fig. 2 ). The slit of the transparent cap was positioned at the forceps hole of the endoscope, where the puncture needle would exit. The endoscope was rotated so that the varix to be punctured was positioned at the slit, and the slit was positioned to pinch the varix ( Video 1 ). Under fluoroscopic guidance, the fixation balloon was inflated and a puncture needle (23G) was inserted into the varix. An assistant injected 0.5 mL of EO under fluoroscopic guidance, and the injection was continued in 0.5-mL increments until sufficient EO had been injected.

**Fig. 2:**
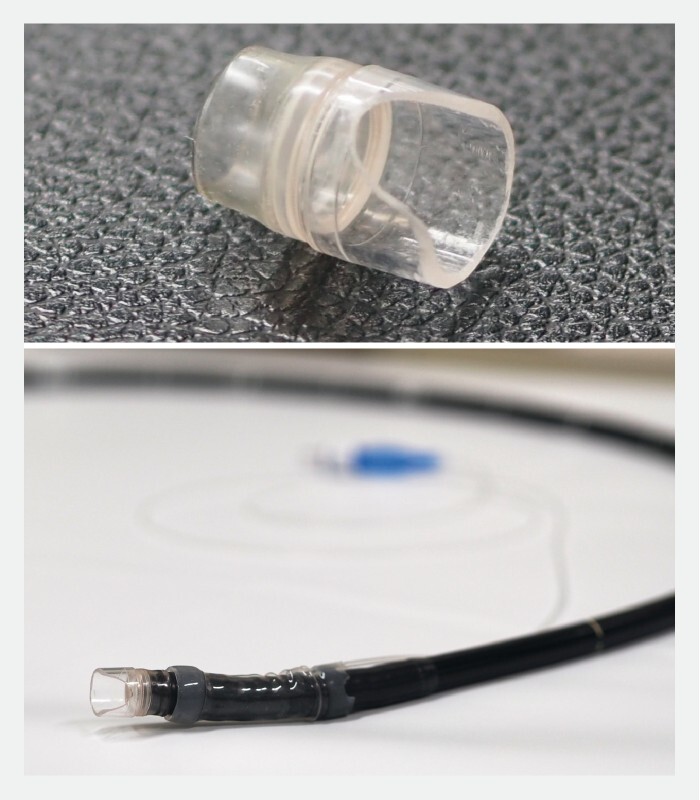
Photograph showing a fixation balloon and the transparent cap with a slit attached to an endoscope.

**Video 1** Endoscopic injection sclerotherapy is performed using the transparent cap with a slit, which facilitates puncture and post-puncture fixation of the esophageal varix.


The transparent cap with a slit facilitates puncture and post-puncture fixation of varices, even when being used by trainees.

Endoscopy_UCTN_Code_TTT_1AO_2AD

## Notes

Endoscopy E-Videos

https://eref.thieme.de/e-videos
*E-Videos* is an open access online section of the journal *Endoscopy* , reporting on interesting cases and new techniques in gastroenterological endoscopy. All papers include a high-quality video and are published with a Creative Commons CC-BY license. Endoscopy E-Videos qualify for HINARI discounts and waivers and eligibility is automatically checked during the submission process. We grant 100% waivers to articles whose corresponding authors are based in Group A countries and 50% waivers to those who are based in Group B countries as classified by Research4Life (see: https://www.research4life.org/access/eligibility/ ). This section has its own submission website at https://mc.manuscriptcentral.com/e-videos
